# COVID-19 Pandemic Experiences of Families in Which a Child/Youth Has Autism and Their Service Providers: Perspectives and Lessons Learned

**DOI:** 10.1007/s10803-024-06402-6

**Published:** 2024-05-20

**Authors:** David B. Nicholas, Rosslynn T. Zulla, Jill Cielsielski, Lonnie Zwaigenbaum, Olivia Conlon

**Affiliations:** 1 Faculty of Social Work, Central and Northern Alberta Region, University of Calgary, Edmonton, AB Canada; 2 Department of Pediatrics, Faculty of Medicine and Dentistry, University of Alberta, Edmonton, AB Canada

**Keywords:** Autism, COVID-19, Psychosocial impacts, Families, Service providers, Service delivery

## Abstract

**Purpose:**

The impacts of the COVID-19 pandemic on autistic children/youth and their families and on service providers are not yet well-understood. This study explored the lived experiences of families with an autistic child and service providers who support them regarding the impacts of the pandemic on service delivery and well-being.

**Methods:**

In this qualitative study, families and service providers (e.g., early intervention staff, service providers, school personnel) supporting autistic children/youth were interviewed. Participants were recruited from a diagnostic site and two service organizations that support autistic children/youth.

**Results:**

Thirteen parents and 18 service providers participated in either an individual or group interview. Findings indicate challenges associated with pandemic restrictions and resulting service shifts. These challenges generally imposed negative experiences on the daily lives of autistic children/youth and their families, as well as on service providers. While many were adversely affected by service delivery changes, families and service agencies/providers pivoted and managed challenges. Shifts have had varied impacts, with implications to consider in pandemic planning and post-pandemic recovery.

**Conclusion:**

Results highlight the need for autism-focused supports, as well as technology and pandemic preparedness capacity building within health, therapeutic and educational sectors in order to better manage shifts in daily routines during emergencies such as a pandemic. Findings also offer instructive consideration in service delivery post-pandemic.

The COVID-19 pandemic was associated with service disruptions that substantially affected autistic individuals and their families, resulting in shifts in daily life. As efforts were made to limit COVID-19 risk, individual and family routines and resources shifted. Public health measures have now largely been lifted, yet some pandemic shifts and impacts remain. This study elicited the experiences of families of autistic children and youth as they navigated the pandemic, from the perspective of both families and service providers.

## Background

Children and youth with autism were deeply affected by the COVID-19 pandemic (Colizzi et al., 2020; Shorey et al., 2021). In terms of daily life, daily routines were interrupted, with early outcomes such as behavioural difficulties and social and communication challenges (Mutluer et al., 2020). School closures limited many supports that autistic children/youth typically access (Colizzi et al., 2020). In one study, over 70% of families of children with special needs experienced decreased educational/therapeutic services (e.g., reduced number of sessions, changes to therapy modality) during the pandemic (Jeste et al., 2020). Virtual delivery of educational and therapeutic services was attempted to sustain services while reducing in-person contact (Assenza et al., 2021; Shorey et al., 2021), with mixed responses to these shifts. Family caregivers expressed concern about the loss of direct involvement of professionals in service delivery, and worried about their child’s skill regression (Latzer et al., 2021; Stankovic et al., 2022).

The implementation of social distancing and infection control protocols in daily life was noted to create substantial confusion for some children/youth. Many autistic children did not fully comprehend the pandemic, as well as related requirements for physical isolation and hygiene/safety precautions. These children/youth variably struggled with adhering to safety protocols (e.g., wearing masks) due to autism-related challenges such as sensory sensitivities (Mutluer et al., 2020). It was perceived that autistic children/youth who understood the pandemic and its implications had better outcomes (Asbury et al., 2021).

### Pandemic Impacts on Families

Changes in therapeutic services and education required many parents to independently support their child’s schooling and support needs (Shorey et al., 2021). The struggle to organize activities amidst a lack of supports (Assenza et al., 2021; Colizzi et al., 2020) reportedly resulted in feelings of inadequacy and anxiety among parents (Assenza et al., 2021; Colizzi et al., 2020; Mutluer et al., 2020). Among families in which the child experienced challenges such as complex behavioral issues, parents reported higher levels of stress related to caregiving, thus seeking respite services (Manning et al., 2020). In comparison to parents of typically developing peers, parents of autistic children reported lower resiliency and poorer coping as well as greater anxiety and depression (Wang et al., 2021).

### Impacts on Service Providers

Similar to what was reported by families, substantial pandemic impacts also were described by service providers. Teachers working with special needs populations indicated that online school platforms impeded their ability to provide multi-sensory curricula (Kim et al., 2021), teach using multiple prompts (e.g., verbal, visual, tactile) (Kim et al., 2021), and support students to self-regulate as they learn (Lambert & Schuck, 2021). Teachers also reported that the delivery of lessons in online school platforms was difficult for children with focus/attention and self-regulation challenges (Lambert & Schuck, 2021). Autism service providers reported concerns related to certain interventions not being as effective over technology platforms, and online platforms not being effective or appropriate with some children, such as those who were minimally verbal (Fell et al., 2023). A lack of technology including devices and internet, and low technology literacy impeded online teaching and assessments (Kellom et al., 2023; Steed & Leech, 2021). Additional challenges included a lack of collaboration with parents in online education (Steed & Leech, 2021), and concerns over the accessibility of online platforms related to English proficiency (Kellom et al., 2023).

The service landscape was drastically modified or decreased (Colizzi et al., 2020), resulting in long-standing impacts that are not yet fully realized. There further has been relatively little examination of how the pandemic was experienced by autism service providers and its impact on their practice. This is a critical gap given that service providers are often relied upon by parents to support and devise strategies to help autistic children. There is need to better understand family and service provider experiences and perspectives relative to gaps and opportunities that have unfolded as a result of the COVID-19 pandemic. To address this gap, this study explored the lived experiences of families with an autistic child and service providers who support them. Research questions were: (1) How was the COVID-19 pandemic experienced by families in which a child/youth has autism, and their service providers?, (2) What were the barriers to, and facilitators of, service delivery in the pandemic?, and (3) What recommendations are offered to improve service delivery?

## Method

Parents of autistic children (to 18 years) and their service providers were recruited from a regional diagnostic/specialty care center and two service delivery centers offering educational, therapeutic and developmental supports within a large city in western Canada. These organizations delivered direct support to autistic children/youth and their families during the pandemic.

Data collection occurred from August 2020 to May 2021. During this period, the jurisdiction underwent several transitions, moving back and forth from restrictions to re-openings in public spaces. Recruitment was undertaken with the support of intermediaries in the above organizations who informed both families and service providers via word-of-mouth and email. Potential participants were provided with information about the study, and if study participation was desired, they were invited to engage in an interview.

Semi-structured interview guides for families and service providers were developed by team members who bring experience in qualitative autism-focused research as well as in service provision, and the team included a parent with first-person family experience of autism. For parents, questions focused on the perceived impacts of the COVID-19 pandemic relative to service access and experience, daily life and well-being. Questions to service providers elicited perceived impacts of the pandemic on service delivery and child, family and service provider well-being. Examples of questions are outlined in Table 1.

**Table 1:** Examples of interview questions

Question (Domain)	For autistic children/youth and their parents	For service providers
Daily Life	What has been your experience during the COVID-19 pandemic?	How did the COVID-19 pandemic impact your work with children/youth?
		How did the COVID-19 pandemic impact your personal life?
Strategies	How did you cope during the pandemic?	What strategies did you use to cope?
Future	Based on your experience, how do you see the future?	Based on your experience in this field, what does the future look like for you?
Lessons Learned/ Recommendations	Based on your experience, what are some lessons that you would like to share?	What are lessons that you learned?

Conducted via Zoom technology or telephone, interviews lasted 0.5 to 1 h. All interviews were audio recorded and transcribed verbatim for subsequent analysis. Families and community service providers who support autistic children/youth and their families were involved in study design, as well as reflecting on findings and manuscript development (e.g., reviewing and providing feedback on the manuscript).

A qualitative content analysis approach guided data management and analysis (Elo & Kyngas, 2008; Graneheim & Lundman, 2004). This approach grounded codes in participants’ perspectives, and delineated experiences, impacts and recommendations. Transcripts were analyzed using a three-step process: (1) reviewing the transcripts to gain an understanding of the data, (2) conducting an inductive analysis, and (3) mapping the data to generate themes (Elo & Kyngas, 2008; Graneheim & Lundman, 2004). The inductive analysis approach comprised three steps: coding data to develop units of meaning *(line-by-line coding)*, grouping these units into a logical schema *(creating categories)*, and interpreting and organizing these units *(determining themes)*. Analysis was undertaken by one team member (JC), with other team members (RTZ, DBN) reviewing a portion of the coding, thus supporting the categorization of codes and generation of themes (Elo & Kyngas, 2008; Graneheim & Lundman, 2004). Reviews of codes, categories and emergent themes occurred on a regular basis amongst team members. NVivo 12 data management and analysis software was used to support the data analysis.

Trustworthiness (qualitative research rigor) was demonstrated via peer debriefing, member checking, referential adequacy and team memoing to document and justify analytic decision making (Lincoln & Guba, 2005). These measures demonstrate thematic resonance with the data and suggest rigor in the analytic process. Institutional research ethics board review and approval were obtained from host university sites prior to study commencement (University of Calgary Conjoint Faculties Research Ethics Board #REB20-0367). Psychosocial support was available and offered to participants if needed, but was not requested. All identifying information was removed from transcripts prior to data analysis.

## Results

In total, 33 individuals participated in this study, comprising 13 parents (12 mothers and 1 father) and 18 service providers. The autistic children of participating parents ranged in age from 8 to 18 years of age, and included 12 males and 1 female. Demographic information about represented families are outlined in Table 2. Service providers comprised healthcare and community service professionals from multiple disciplines (e.g., physicians, psychologists, speech language pathologists, behavior analysts, occupational therapists, social workers, nurses), school-based providers (e.g., educational consultants, teachers) and program administrators/leaders (e.g., program coordinators, program/agency directors). All healthcare/service providers worked in autism-related services and practiced during the pandemic.

**Table 2:** Family characteristics

Characteristic	*N*
Ethnicity	
Caucasian	5
European	2
South American	1
African	1
South American and Canadian	1
First Nations and European	1
Caucasian, East Indian and Jamaican	1
European and Canadian	1
Home Region	
Urban (metropolitan area > 80,000)	10
Suburban (within 1 h commute to urban area)	1
Rural (< 20,000 over 1 h from metropolitan area)	2

Overall, participants stated that autistic children and their families experienced significant challenges and adjustments on a daily basis during the COVID-19 pandemic. Parents navigated various services in seeking to help their child—some with perceived benefits and others not. Service providers described seeking to accommodate children’s needs while adhering to shifting safety management protocols. Themes emerged in each group (families and service providers), as follows: (a) pivoting quickly and on an ongoing basis, (b) accommodating changes and addressing needs, (c) impacts on health and well-being, and (d) holding onto hope yet concern for the future. The themes include sub-themes, as outlined below.

### (a) Pivoting Quickly and on an Ongoing Basis

The onset of the COVID-19 pandemic elicited rapid shifts in services, with deleterious impacts on families and service providers. Emerging sub-themes within this experience were ‘Service Shifts and Uncertainties’ and ‘Family Fears and Strains’; each sub-theme is outlined below.

#### Service Shifts and Uncertainties

Services that were deemed ‘non-essential’ (e.g., schools, accessible transportation services) were closed or substantially decreased or modified, resulting in limited resources and support for autistic children/youth and their families, notably with some services shifting to virtual modalities. Service providers needed to rapidly seek to understand and then navigate resources to address child/family needs. As one service provider recalled,

*Non-essential services were closed and essential services were open, and we felt like we fit in a middle ground there. We really needed to figure out how to continue to provide services to our families. We knew the families were going to be really struggling with kids out of school and out of routine. But we weren’t necessarily designated as an essential service in an obvious way. So we had to establish a connection… so that we were able to continue to provide services, and that gave us that opportunity to do a critical support needs assessment so we could focus our attention on those [children/families] with the highest level of need.*

Service providers felt that the lack of clarity about what constituted an ‘essential’ service in the pandemic, left families unclear about what they could access which further left them under-served. Service insufficiency and vagueness in guidelines heightened the demands of family caregiving, as observed by a service provider:

*It was so unclear to families [whether] they were [or were not] allowed to have respite [care]. Of our families, at least 50% of them stopped having anyone come into their home so now they have twenty-four hours a day [caring for] their child with huge needs, and they’re in charge twenty-four hours which made them burn out.*

Due to public health protocols, in-person supports were transitioned to an array of remote/electronic formats (e.g., telephone, text, email, online synchronous platform). For many service providers and families, this change required rapid learning about how to use various online platforms and navigate shifting services. A service provider recalled,

*We shifted gears… overnight. We went from having no video conference capacity to here’s your Zoom login and ID, and then we all had to figure out how to do a Zoom meeting.*

Service providers described trying to prioritize support needs such as addressing behavioral struggles and emotional strain due to the loss of school and diminished autism-related supports. They recalled seeking to implement service flexibility to better accommodate family needs and schedules. Yet, they also described uncertainty and worry as they adjusted quickly amidst unknown health risks associated with these shifts.

Making changes in service delivery was described to be especially difficult early in the pandemic. For service providers, modifying services to align with safety protocols was rendered more challenging due to a lack of knowledge about COVID-19, uncertainty about the effectiveness of safety protocols, and unknown impacts of these shifts and service gaps on autistic children and their families. A service provider recalled,

*I never said, “It’s going to be okay” because I didn’t know. But I would say, “We’re going to figure this out.” And then I’d wait for direction, but the communication was not there…. No one knew what the heck was going on… so that fear of the unknown was very, very present with the team.*

Shifting services became particularly difficult in cases in which children/youth required assistive and/or medical support. For instance, one service provider recalled significant challenges coordinating and sending communication devices to families in distant rural regions during service and school closures. Seeking to offer support in these circumstances, staff improvised and sought to remain in relatively close contact with families, as possible, as reflected on by a service provider:

*When COVID-19 first started, we gathered information from the families and then prepared documents of a whole bunch of things to share with families. As a coordinator, I went to every single family’s home and whether I stood outside or at their kitchen table, whatever they were comfortable with and had conversations about COVID-19, about impact, [and] about what our services were to look like.*

#### Family Fears and Strains

For families, the pandemic ushered in fears about COVID-19 transmissibility as well as decreased services to address anxiety and support well-being. An array of services (e.g., in-home support, recreation, social skills, healthcare, public transportation) were either shut down, modified, or delayed. For instance, alterations to public transportation meant that some families had to delay appointments. The sudden shutdown of schools and after-school resources was described as ‘*terrible*’, ‘*so stressful*’ and ‘*very confusing*’ by families. Parents sought to help their autistic child accommodate to sudden transitions in school and after-school activities, yet children/youth had significant difficulty, as illustrated by a parent who reflected on the difficulty with school transitions:

*School was quite difficult. It was really difficult to get [autistic child] to do the school [work]…. He was supposed to have… two hours of work, but… it was taking him like four hours…. It was a big stress on our relationship too because… I was trying to get him to do the work and he didn’t want to.*

Changes in daily routines such as requisites to stay home, were difficult for some children/youth to understand and accept. In response, parents trialed various strategies to help their child cope. For instance, some parents created a schedule for their child in an attempt to heighten predictability in the day and week.

### (b) Accommodating Changes and Addressing Needs

As the pandemic continued over time, families experienced ongoing changes in service delivery and in daily routines. Sub-themes in this overall theme were, ‘Prioritizing Safety, but at a Cost’, ‘Reliance on Virtual Services’, and ‘Family Adjustment’, as described below.

#### Prioritizing Safety, but at a Cost

As the COVID-19 pandemic lingered, the need for adherence to safety and adjustment to ongoing changes remained a priority for agency leaders as they sought to support families relative to child development and well-being. Service providers described extensive measures such as the use of personal protective equipment and social distancing, while simultaneously monitoring and attending to child/youth and family needs. With a focus on safety, service providers felt hindered in their ability to effectively engage children/youth as they were accustomed to, and thus provide optimal support, as described by a service provider:

*We can’t really do our jobs [when we’re] six feet from our kids [service recipients] because in order to actively engage them and get them interested in what we’re doing, I can’t be across the room right, because often times, they thrive upon like facial expression. We’re interacting with something and we are modelling what to do with it. Well, if I’m sitting on the other side of the room…, they’re not going to be engaged with me.*

In terms of economic sufficiency and stability, both service providers and families variably faced vulnerability. Compensation for some service providers and parents were viewed as inadequate and difficult to access. Reflecting on required employment absence due to illness or quarantine, a service provider commented:

*The funding to cover you when you are sick or you have to isolate which is a government mandate, only covers you one time a year…. In this global pandemic, they’re saying people are going to go on multiple bouts of isolation…, but we provide support [only] once. How are businesses supposed to keep up with that? They can’t. But how are people supposed to go on isolation multiple times without getting paid?*

Negative pandemic impacts on financial stability both for families and services providers were deemed to be under-addressed. Further, inflationary pressures later in the pandemic resulted in lingering financial strain, with potentially heightened and continuing effects on families and service providers.

#### Reliance on Virtual Services

As the pandemic persisted, service providers modified supports through a combination of in-person and virtual formats (e.g., email, online, telephone, synchronous platforms). In choosing approaches, families’ needs and preferences, to a degree, guided services, as noted by a service provider:

*The families became the driver… because they were in it twenty-four hours a day and they could see, ‘here’s what we need to work on’. It was less ‘cookie cutter’ service and more family-focused.*

Virtual service delivery was seen to have benefited service providers through eliminating the need to travel to and from appointments, providing a venue for quick check-ins with families, and offering an efficient means for coworkers to meet and engage in peer support. For several families (but not for all), virtual support emerged as a vicarious *silver lining* of the pandemic in advancing an alternative platform for service delivery to children/youth and families. A service provider noted,

*[Technology] opened up the way we communicate. Now our staff is asking our participants, “What is your preferred way of communicating? Should I text you? Should I FaceTime you? Are we emailing? Do you like phone calls?” So we’ve learned to actually expand the way we communicate with our participants, and it’s been extremely positive. Moving forward, we are trying to find ways to continue that because our participants are even wanting to communicate with each other which has never come up before, but now they are looking. It’s almost like they’ve found a new way that’s acceptable to communicate.*

While identifying benefits of online communication, the adjustment to the use of virtual platforms was viewed both positively and negatively. Service providers contended that success in transitioning to and using online support platforms with families seemed to depend on both the age of the child and the quality of relationship between the child/youth and service provider. Service providers indicated that younger children tended to have more difficulty transitioning to online social support groups relative to older children/youth who, in many cases, were more comfortable engaging in online dialogue. Transitioning from in-person to virtual support was deemed to be easier for families with whom service providers had a pre-existing relationship and strong rapport, as opposed to families that accessed service provision after the introduction of restrictions to face-to-face contact.

Along with benefits, service providers noted challenges with using online support platforms. These included communication barriers, individual/family lack of access to or comfort with technology, overuse of technology by children/youth, lower levels of interest/participation among families, and online fatigue. Such challenges created uncertainty among service providers about the utility of virtual support for all families and circumstances. In such instances of suboptimal or negative impacts of technology for support provision, service providers doubted their capacity to do their job as well as they could have in person. As an example, a service provider described a sense of helplessness intervening via virtual engagement:

*Sometimes you would be watching a kid literally slamming their head on a wall and you can’t do anything because you’re on this side of the computer. Sometimes it was just about sitting with the parents and saying, “I see everything, [and] you’re [doing] a very good job…. You’re being everything your child needs you to be right now. This is hard and I see you and I hear you”. It was really hard to not be that person that could be in the home with them. Our service model is to be in there, doing the ‘nitty gritty’ with the parents, and working through it together.*

Another service provider lamented about the shift from face-to-face to virtual programming:

*Every [identified evening each week] was this party that everybody went to. I have been doing this for fifteen years and I always look forward to [that] night, and I would just love going around the recreation center, and seeing all the social skills groups and the parents looking so happy. Here’s all this range of parents meeting in their very normal environment, and having coffee and tea and it’s all support. I think that was a big loss for me personally as a clinician.*

#### Family Adjustment

Adjustment to the pandemic was experienced in varying ways by individuals and families. Most parents indicated that their child adjusted to required protocols such as masking, social distancing and washing hands, though some indicated the need for prompting and/or assistance. In a few cases, parents reported needing to continually provide guidance and/or reinforcement to ensure their child’s compliance with safety protocols. Several parents further stated that their child’s difficulty adhering to protocols resulted in decreased outings and in turn, greater social isolation:

*We don’t take [the autistic child out of the home] anymore. He doesn’t do really well with masks. I don’t want to risk tak[ing] him out and then he will have a meltdown.*

The prolonged duration of the pandemic was reported to require ingenuity and persistence by parents in seeking strategies to meet their child’s pressing, and in some cases, shifting needs. Some parents described organizing “safe” social gatherings (e.g., socially distanced activities), and strategically setting up occasions for social connection, as needed, to support child/youth wellness. One parent utilized increased time at home during the pandemic to teach her child household skills such as cooking, dishwashing, house cleaning and laundry.

To help manage and cope with additional family caregiver tasks and strains, parents variably relied on government resources (as available), and in some cases, used these resources to fund needed supports. While such resource availability differed among families, support sometimes was provided by relatives, neighbours, privately-funded services and/or other resources in the community. Learning to be independently resourceful and a sole (or predominantly sole) caregiver during the pandemic was consistently described as difficult, and was especially so for those who had previously relied heavily on formal supports (e.g., therapeutic services, respite care) to address the daily care needs of their child and, in particular, those whose child had complex care needs.

Periodic shifting from in-person to online school (and vice-versa) created substantial difficulty for some children/youth and families. Despite these challenges, additional support was described by several parents as largely unavailable. Parents described a range of responses including both appreciation and frustration/dissatisfaction with modified services and schooling. As an example, a parent conveyed appreciation; however, this reflected challenge with online schooling:

*He (the child) would get up and once he saw her (the school attendant’s) face, she (the school attendant) would get him out of bed. [Otherwise] he wouldn’t get out of bed [so] I’d have the phone right there. She was amazing. Now I don’t know if that was something that the school had told her to do. I really believe it was something that she had done all herself. I really believe it was an individual thing, same with all of [her support with] the curriculum…. I’m pretty sure she modified everything herself, with no support or guidance from any professional from the school.*

When online schooling transitioned to in-person delivery, some parents chose to continue online often to avoid heightened risk of their child acquiring COVID-19. In other cases, parents sent their child to school in-person; however, with concern about risk of exposure to COVID-19, as recalled by a parent:

*People started testing positive in the schools and then the unknown. So then I had to take him out of it, and keep him home because he was getting anxious and frustrated.*

### (c) Impacts on Health and Well-being

Health and mental health impacts were experienced by both family members and service providers as a result of the extended existence of pandemic conditions and the rapid, multiple and ongoing changes in daily routines and service provision. The sub-theme, ‘mental distress of service providers’ highlights professional and personal tensions for service providers particularly as they navigated pandemic restrictions and the sub-theme, ‘psychosocial and behavioral impacts on families’ reflects challenges experienced by autistic children/youth and their families. A final sub-theme, ‘positive adaptation’ identifies areas of growth or positive experiences associated with the pandemic, as well as resources for moving forward. Each subtheme is described below.

#### Mental Distress of Service Providers

Service providers conveyed professional and personal tensions such as worry and feelings of disconnectedness from family, friends and co-workers while ‘putting on a brave face’ and supporting families. A service provider reflected on her ongoing adjustment and struggle with shifting public health guidelines:

*The changing of the guidelines [was tough]. One week we think we have got it, we’ve got everything laid out, we have a plan. And then we come back on the Monday and the guidelines have shifted again…. The fatigue of new kinds of policies and procedures always crop up. Then you are trying to remember what the latest one was, and hope that you are ticking off all of the boxes, you are doing things correctly…, not forgetting something or being negligent.*

Perceived burden from these shifts and tensions increased at key junctures of the pandemic. For instance, when schools closed, educational assistants experienced fear of financial insecurity and job loss. Once schools opened, educational assistants experienced concern as they returned to work yet had to quarantine on a frequent basis and were worried that COVID-19 sick leave benefits would not cover living expenses.

Over time, service providers reportedly became more familiar with, and adept at adapting to, a changing environment. Administrators variably sought to develop organizational or team strategies to support their employees. Strategies included mental health/wellness checks and support to staff in an attempt to create safe spaces to debrief and provide emotional support, exchange strategies, trade work tasks, facilitate peer support and cover shifts for fellow employees.

The ambiguity of what constituted “essential” services was reported to be stressful and problematic for parents and service providers. Of particular concern, unclear understanding about ‘vulnerable’ communities created misperceptions and under-recognition of the unique daily challenges that families of autistic children/youth experienced. For several parents, pandemic-related challenges were conflated with other struggles, as described by a parent:

*I think they should realize that the special needs population, kind of like the elderly population, isn’t going to have the same issues that the general population has. Everything is like more emotional, more exacerbated when you have this cognitive gap in understanding, you have a gap in physical support because you can’t be around people. Life is very isolating when life is normal, right? We can’t go to the same places and do the same things that other families do when life is normal. But then you add a pandemic on top of that so now you’re even more isolated, and it’s very difficult. I mean I’m sure this happens to all the parents, but you burn out and [elements of this circumstance aren’t] going to get better over time.*

#### Psychosocial and Behavioral Impacts on Families

Parents observed heightened child/youth anxiety, loneliness and challenging/problematic behaviors (e.g., meltdowns, chewing, picking, aggression) during the pandemic. The loss of in-person educational, recreational and social activities (e.g., school, music lessons, swimming instruction, summer camps) meant a lack of opportunities to socialize with peers and engage in desired activities, as reflected by a concerned parent:

*Our son has been struggling with aggression…. At the beginning of the COVID [pandemic], it was… bad and then it… got a bit better…. We fell into a bit more of a routine and stuff. And then the last couple of months,… he’s been very aggressive. So my anxiety is really bad now because every time he moves towards me, I’m worried that he’s going to hurt me.*

Parents reported substantial stress from the disruptions in services, and the resultant pressure to find alternative resources to support their child’s pre-existing, emerging or heightened needs. Some described worry about preserving their existing supports, and were uncertain about how to navigate autism service funding during the pandemic, with further concern that if their funding allotment (from government-provided autism supports) wasn’t spent, it would be lost. Learning to support their child in multiple areas and managing personal/family pressures (e.g., managing schooling, supporting their child’ adjustment, behaviors, emotions and socialization, dealing with strained finances and family pressures) grew increasingly difficult, and yielded emotional burden on parents. This was ascribed as an intense pressure and sense of personal failure by a parent:

*I felt like I was failing as mom, I’m probably going to get emotional, I felt awful. I was so stressed out and my husband’s hours at that point, still hadn’t been cut, he was still working regular hours and so it was like this is bogus. Like I can’t do this. Me having [a learning disability] and having to teach my kids. My son’s teacher and I weren’t getting along so some of her emails [that were] coming back at me were [critical of me]. I had to send them an email saying, “For my mental health right now, I cannot have to feel the pressure of having my kid’s school work done, because these marks that you are giving them are reflecting me. It is making me very depressed right now. I can’t handle this, no!” They’re sitting there telling my kids they’re doing their stuff wrong. Well, it’s not them doing it wrong, obviously it’s me teaching them wrong because I don’t know what you’re really asking.*

Along with care-related transitions and struggles, some parents identified relational challenges within family life. Relational challenges included marital conflict and difficulty coping with strain that was exacerbated by reduced supports.

#### Positive Adaptation

In contrast to these challenges, positive experiences were also described by some participants. For several parents, the changes in service delivery did not have as drastic and negative an impact on their children as they had anticipated. Several parents reported not experiencing medical support disruptions or adverse health impacts, and some perceived improved well-being in their family. Similarly, some parents identified no or few deleterious impacts on their child’s social skills or mental health. Two parents felt that online school enabled their child to maintain helpful connections with teachers and peers.

Supporting adaptation and coping during the pandemic, parents described the buffering effect of optimism, skill development (e.g., navigation), and supportive relationships. Prior to the pandemic, several parents had an established network of support which they felt had continued and ultimately contributed to their own and their family’s well-being in the pandemic. These resources were viewed to be helpful, as illustrated by a parent:

*As soon as the school stopped running, everybody lost their occupational therapist, physical therapist, behavioural therapist, their sensory rooms—everything. Families were struggling and I was very lucky… because I’ve been doing this for a little while and reaching out and building up my toolbox.*

Several parents described appreciation for dedicated and compassionate service providers (e.g., teachers’ aides, community service providers, government resource workers) with whom they worked. For instance, several parents recalled teaching assistants providing extra time and support to their child, which was deeply valued.

### (d) Hope Yet Caution for the Future

Support for families and service providers was noted to positively impact their expectations for the future. Such beneficial support was categorized in sub-themes of, ‘targeted supports’ and ‘ongoing supports’, with each sub-theme briefly summarized below.

#### Targeted Supports

Both parents and service providers recommended targeted pandemic-related supports to families. Parents suggested resources for children/youth such as explanatory social narratives or focused conversations to help autistic children/youth better understand appropriate detail about a given disaster (e.g., a pandemic) and resulting protocols. They suggested that service providers need to be empathic toward parents and families, and parents need to be patient with themselves and their children. Service providers emphasized their need to incorporate self-care, and they suggested that policy/program-based decision making in community care should better address and reflect the needs of workers, as illustrated by a service provider:

*I think one of the main pieces of work that really helped us and really drove us forward and has held us steadfast today, six months later, and I would recommend that any organization going through anything similar do this in the future, is to develop a set of principles by which to make decisions and spend time on that. We were able to develop about five principles and we have gone back to that several times. We had a lot of input from the staff, it’s very much a shared development of those principles so that is a recommendation because I think that really helps to figure out what’s most important for you as an organization and how to make decisions.*

Recommendations for policy and decision makers focused on clearer messaging and enhanced capacity to support families of autistic children/youth. Parents emphasized the need for timely and tailored communication regarding required service delivery changes. They further advocated for additional funding and the availability of respite services and after-school activities for children/youth. These changes were thought to more likely result in ensuring needed resources in constrained conditions such as a pandemic. Service providers suggested revisiting and widening the definition of ‘essential’ services to incorporate autism services. All participant groups emphasized the need for heightened pandemic planning and capacity building among education and service organizations in supporting autistic children/youth.

#### Ongoing Supports

In moving forward, several parents and service providers hoped for continuing online supports during and beyond the pandemic. One parent desired access to online communication resources within daily school routines. Several service providers recommended an expansion of virtual service delivery during and after the pandemic in the aim of minimizing the need for travel to services by families or, in the case of home visits, time required of services providers for travel. Yet for other service providers and families, the return to face-to-face services was strongly welcomed; hence, ongoing flexibility of service delivery modalities has emerged as important.

## Discussion

This study elicited the experiences of families with an autistic child and their service providers as they navigated the pandemic. Findings demonstrated substantial shifts including a protracted reduction and modification of therapeutic and educational supports during the pandemic. Findings amplify important areas for consideration that are both specific to pandemic conditions *and* extend beyond this historical ‘moment’ of pandemic constraint.

Decreased and modified services in the pandemic left parents pressured to independently fend for themselves in accessing support for their child and family. Such a shift and challenge corroborate earlier studies reporting deleterious impacts on autistic children/youth and their families as a result of service shutdowns and reductions (Colizzi et al., 2020; Garcia et al., 2021; Mutluer et al., 2020; Wang et al., 2021).

Coping mechanisms via personal assets (e.g., acquiring multiple skills, knowledge of resources) and networks of support were integral to navigating the pandemic. This finding is aligned with studies that convey the tenacity and proactivity of parents in: (i) educating their children on the emerging daily realities of COVID-19 (Majoko & Dudu, 2020), (ii) creating home-based child/youth learning (Majoko & Dudu, 2020), and (iii) building a network to support caregiving and wellness (Majoko & Dudu, 2020). Study findings indicate the need to develop educational interventions to enhance family caregivers’ skills (e.g., wayfinding, advocacy), and to ensure service sufficiency (e.g., respite care).

Studies further document multiple challenges among service providers such as moving from in-person to virtual services (Sheehan et al., 2022; Spain et al., 2021), shifting service delivery options (e.g., forgoing lengthy diagnostic assessments; Spain et al., 2021), adhering to infection control protocols (Sheehan et al., 2022), and configuring care spaces (e.g., adhering to public health protocols; Sheehan et al., 2022; Spain et al., 2021). For service providers, stress emerged due to perceived risk to personal and family safety (Sheehan et al., 2022), which invites human resource policy and practice planning within the disability sector. Other priorities include support for work-life balance and capacity building in the optimal use of virtual technology (Sheehan et al., 2022).

Similar to existing literature, this study illuminated stretched workloads of community and educational service providers due to the extensive support needs of children/youth and families amidst heightened stress and constrained resources. Pre-existing system gaps (e.g., funding cuts, insufficient services) and new pandemic-related challenges (e.g., insufficient financial compensation for quarantine leave, lack of time to adjust to new operational protocols), coupled with ongoing uncertainty (e.g., unknown health risk, unpredictable shifts in public health guidelines) culminated in extraordinary burden, with physical and mental health impacts. Ongoing shifts over the course of pandemic, changes in daily work life and deleterious impacts on worker well-being, have required agencies to pivot over the course of the pandemic, yet with limited alternative staffing options. This in turn is purported to have resulted in continuing strain among service providers. This strain on the well-being of services providers post-pandemic and in future emergencies, warrants further inquiry and consideration of human resource support needs.

Study findings illuminate the need for technology capacity building in autism services. Transitioning from in-person to virtual services and education requires re-configuring complex content, with relational and environmental considerations that may not easily translate across these diverse service delivery/teaching modalities. Best practices are needed to optimally support children in their development at difficult times and contexts such as pandemics or other disasters. In moving forward, changing norms regarding flexibility in service delivery approaches and the viability of virtual options for some children and families may provide ongoing opportunities to enhance care, given the positive experiences described by some of the participants in the current study.

In terms of schooling within a pandemic, collaboration between parents and teachers seems integral to supporting children/youth (Kim et al., 2021; Schuck et al., 2023). While the literature does not highlight the extent or role of collaboration prior to or during the COVID-19 pandemic, collaborative relationships appear pivotal in helping to address children’s challenges within and beyond a pandemic, and in proactive planning for a possible future widespread emergency/disaster.

Experiences from the literature and this study inform virtual support development for children/youth and their families. Although virtual modalities to support families emerged in the 2000s (Kane & DeBar, 2023), such remote services exponentially increased in the COVID-19 pandemic, as exemplified in this literature (Fell et al., 2023) and our study. Building a strong technology infrastructure (e.g., accessibility, effectiveness metrics) is needed. Studies addressing virtual interventions for autistic children/youth have highlighted the importance of providing training to implementers (Fell et al., 2023; Kane & DeBar, 2023), tailoring the intervention to the individual (Bundy et al., 2023; Fell et al., 2023), ensuring access and capacity to use online platforms (Fell et al., 2023; Kane & Debar, 2023), and infusing infrastructural and administrative supports to sustain the intervention (Fell et al., 2023). Additional considerations highlighted in this study include the importance of wrap-around technological support particularly in the early stages of one’s use of an online platform and incorporating diverse methods to engage families. Attention to the social determinants of health and upholding an equity and inclusion framework are critical in equitably supporting the range of population needs and addressing variability in technology access (Zwaigenbaum et al., 2021). Further, integrating an equity lens in the distribution of resources is critical to moving forward in a socially just manner.

Preparedness for a pandemic or other disaster is integral to supporting autistic children and youth and their families. In this study, accessible information tailored to the specific needs of children, youth and families was strongly recommended. Organizational and systemic strategies are needed to ensure such information and service coordination are available (Dai & Hu, 2021; Kusumi et al., 2023). As an example of such an approach, crisis emergency teams were created within a special needs school in Japan to provide ongoing guidance throughout the COVID-19 pandemic. These teams comprised school staff and health authority personnel (Kusumi et al., 2023). Such innovation illustrates important collaboration across sectors to develop responsive strategy. This is particularly important given that specific communities (e.g., the autistic community in a rural and remote region) may have particular logistical, informational and support challenges (e.g., impeded information and resource access).

Targeted COVID-19 information and supports for the autism community likely would have assisted autistic individuals and their families to better navigate the pandemic. Systemic vagueness in determining ‘essential’ services and the determination of some key services as ‘non-essential’ rendered it challenging for therapeutic and educational personnel to work with autistic children/youth and their families as they otherwise likely would have. These findings amplify the importance of clear messaging focused on pressing issues, with attention on the needs of autistic children and their families.

The literature is beginning to document disability-led initiatives and innovation that addressed informational gaps in the pandemic. For instance, in China, a disability-led initiative emerged from growing concerns that families of children with developmental disabilities required appropriate and updated information on COVID-19 risk, strategies to mitigate COVID-19 exposure, and supports for children. This initiative comprised international and local volunteers who (i) tailored and disseminated comprehensive and inclusive web-based COVID-19 information (e.g., health risk and prevention strategies) to disability communities, and (ii) coordinated the delivery of tangible resources (e.g., medication, PPE) to families (Dai & Hu, 2021). In Canada, AIDE Canada (www.aidecanada.ca) offered a devoted website with COVID-19 resources specific to autistic individuals and their families. Strategies and resources addressing pressing issues for autistic individuals and their families emerge as priorities in the event of a potential future disaster such as a pandemic.

Overall, these findings offer important considerations for critical reflection and service advancement that go beyond the COVID-19 pandemic (Zwaigenbaum et al., 2021). For instance, service insufficiency and systemic challenges have persisted, and warrant critical reflection and targeted action. Post-pandemic mental health concerns, financial challenges among families, and deep resource and service gaps, are examples of continuing areas of challenge to be addressed.

### Study Limitations

While addressing the experiences of families and service providers, this study did not specifically gather perspectives from school staff and residential care workers who supported autistic children/youth during the pandemic. The sample lacked extensive first-person experience from autistic children/youth, and disproportionately included families in which the autistic child/youth was male; hence, further study is needed based on a broader sample representation. The sample further reflected lived experience in only one region in Canada which highlights the need for future studies within broader regional and sociodemographic contexts in ascertaining how regional and other demographic factors may nuance service delivery experiences.

Study data were collected relatively early in the pandemic; hence, emergent or longer-term impacts of the pandemic (and beyond) are needed, as are future interventional studies that examine proactive approaches fostering preparedness, pandemic support and post-pandemic recovery. Notably, such additional research is underway by members of this research team.

## Conclusion

This study strongly asserts the need for pandemic and post-pandemic planning and support, with specific focus on the needs of autistic children and their families. To that end, collaboration amongst those with lived experience in the autistic community, service providers and program/policy decision makers is integral in the important aim of ensuring sufficiently-resourced supports and services both within and beyond a pandemic.
